# Artifact Rejection Methodology Enables Continuous, Noninvasive Measurement of Gastric Myoelectric Activity in Ambulatory Subjects

**DOI:** 10.1038/s41598-018-23302-9

**Published:** 2018-03-22

**Authors:** Armen A. Gharibans, Benjamin L. Smarr, David C. Kunkel, Lance J. Kriegsfeld, Hayat M. Mousa, Todd P. Coleman

**Affiliations:** 1 Department of Bioengineering, University of California at San Diego, La Jolla, CA United States; 2 Department of Pediatrics, University of California at San Diego, La Jolla, CA United States; 3 Department of Psychology, University of California at Berkeley, Berkeley, CA United States; 4 GI Motility & Physiology Program, University of California at San Diego, La Jolla, CA United States; 5 Neurogastroenterology and Motility Center, Rady Children’s Hospital at San Diego, San Diego, CA United States

## Abstract

The increasing prevalence of functional and motility gastrointestinal (GI) disorders is at odds with bottlenecks in their diagnosis, treatment, and follow-up. Lack of noninvasive approaches means that only specialized centers can perform objective assessment procedures. Abnormal GI muscular activity, which is coordinated by electrical slow-waves, may play a key role in symptoms. As such, the electrogastrogram (EGG), a noninvasive means to continuously monitor gastric electrical activity, can be used to inform diagnoses over broader populations. However, it is seldom used due to technical issues: inconsistent results from single-channel measurements and signal artifacts that make interpretation difficult and limit prolonged monitoring. Here, we overcome these limitations with a wearable multi-channel system and artifact removal signal processing methods. Our approach yields an increase of 0.56 in the mean correlation coefficient between EGG and the clinical “gold standard”, gastric manometry, across 11 subjects (p < 0.001). We also demonstrate this system’s usage for ambulatory monitoring, which reveals myoelectric dynamics in response to meals akin to gastric emptying patterns and circadian-related oscillations. Our approach is noninvasive, easy to administer, and has promise to widen the scope of populations with GI disorders for which clinicians can screen patients, diagnose disorders, and refine treatments objectively.

## Introduction

Gastrointestinal (GI) problems are the second leading cause for missing work or school in the US^1^, giving rise to 10% of the reasons a patient visits their physician, and costing $142 billion annually^2^. Symptomatic management is routinely used by primary care physicians, and patients are referred to GI specialists if symptoms persist, which happens most of the time^2^. While pathologic findings can be detected with a blood test, endoscopy, or imaging, oftentimes symptoms cannot be attributed to a medical condition despite appropriate workup. These disorders fall under the umbrella of functional and motility GI disorders and make up a majority of patient referrals to GI specialists. Examples include: functional dyspepsia^3^, which is reported by up to 20% of children and adolescents^4^ and is predicted to affect 20–40% of all the US population at least once in their lifetime^3^; and gastroparesis, characterized by delayed gastric emptying in the absence of a mechanical obstruction, which affects 4% of the US population^5^, including 70% of Parkinson’s patients^6^ and 50% of diabetes patients^7^.

Functional and motility GI disorders are typically diagnosed with subjective symptom-based assessment or objective, but invasive, intermittent procedures in specialized centers. Symptom-based diagnosis is problematic because these disorders typically have overlapping symptoms but different treatment regimens^8^. Antroduodenal manometry, a procedure that measures motility with a catheter inserted through the mouth or nose with fluoroscopic or endoscopic guidance, can differentiate between myopathic and neuropathic disorders and can change the diagnosis and treatment of 15% to 20% of patients with upper GI symptoms^9,10^. However, it has drawbacks of long wait times, cost, and invasiveness. That these procedures can only be performed intermittently in specialized clinical facilities is also at odds with the transient nature of functional and motility GI disorders, making inconsistent events difficult to detect. The GI tract is affected by stress^11^, and thus also susceptible to “white coat syndrome”, where patients experience increased anxiety with being in the clinic^12^. Between the physical and psychological discomfort, there is a strong preference by patients for noninvasive and ambulatory testing instead of current approaches^13^. Altogether, these GI disorders are not routinely diagnosed, result in adverse social and psychological outcomes, and place tremendous pressure on GI specialists^14^.

Functional and motility GI disorders are broadly comprised of pathophysiological disruptions of normal GI motility, which give rise to symptoms including abdominal pain, nausea, bloating, fullness, and early satiety^15,16^. GI smooth muscle contractions are initiated and coordinated by underlying rhythmic bioelectrical patterns, termed slow-waves. In healthy individuals, gastric slow-waves are generated three times per minute (i.e., 0.05 Hz) and propagate along the stomach’s longitudinal axis towards the small intestines^17^. These depolarization waves are coordinated with ring-like smooth muscle contraction waves (i.e., peristalsis) that physically break down food and propel it down the GI tract. Since the myoelectric patterns in patients with disrupted motility are altered compared to those without GI symptoms^18,19^, it follows that extraction of these patterns has the potential to improve screening tools, diagnoses, and treatment regimens.

Gastric myoelectric activity can be measured noninvasively using skin-mounted electrodes, referred to as the electrogastrogram (EGG), traditionally recorded using a bipolar configuration of electrodes to produce a single channel measurement^20–23^. Unlike the electrocardiogram (ECG; recordings of heart electrical activity), the EGG has not seen widespread clinical adoption due to its poor correlation with gastric emptying tests, manometry, and diagnosed disease status^24^. These unsatisfactory results arise in part from inconsistent and poor signal quality^25^. ECG signals are strong (i.e., 1–3 mV) and contain well-defined, repeating motifs (e.g., the QRS complex) that lend themselves to quantitative and qualitative analysis. In contrast, EGG biopotentials are weak, generally within the range of 50–200 μV. Therefore, the signals must be significantly amplified, which increases the likelihood of noise and artifacts occluding the signals of interest.

Motion artifacts in the EGG signal are abundant enough in clinical settings to make EGG difficult to interpret^24^. Furthermore, they occur so often in ambulatory settings that there is currently no clinical solution for recording ambulatory EGG, which would capture information during intermittent GI symptoms in non-stressful environments^26,27^. The negative impact of these artifacts is magnified by standard EGG analysis, where data is typically binned into several-minute windows for spectral analysis of slow-waves. As such, even short-duration artifacts can render large time periods uninformative by introducing broadband power in spectral estimates^25^. Previous methods of removing artifacts from EGG recordings either delete segments of data^28^ or are computationally inefficient^29^, both of which cause challenges for the large datasets generated by ambulatory recordings.

Much can be learned from artifact removal in electroencephalogram (EEG; recordings of brain electrical activity) due to its similarly low amplitude, but the location of the electrodes on the torso along with the low-frequency content of the EGG signal presents unique challenges. Stereotyped artifacts (i.e., their origin remains in the same location in the body), such as eye movements and the cardiac electrical signal, dominate the EEG^30^. On the other hand, non-stereotyped artifacts from abdominal and cable movements are the main concern for EGG recordings^28^. While most stereotyped artifacts occur at higher frequencies and can be removed from the EGG by filtering techniques, motion artifacts span across all frequencies and therefore filtering alone does not suffice. Independent component analysis (ICA), a popular approach for removing artifacts from the EEG^31^, is not ideal for removing non-stereotyped artifacts due to the unique spatial patterns on the recorded waveforms that adversely affect ICA decompositions^30^. In practice, these types of artifacts are manually removed prior to ICA analysis^32,33^; such manual intervention limits the ability for widespread automated analyses in ambulatory recordings.

**Figure 1:**
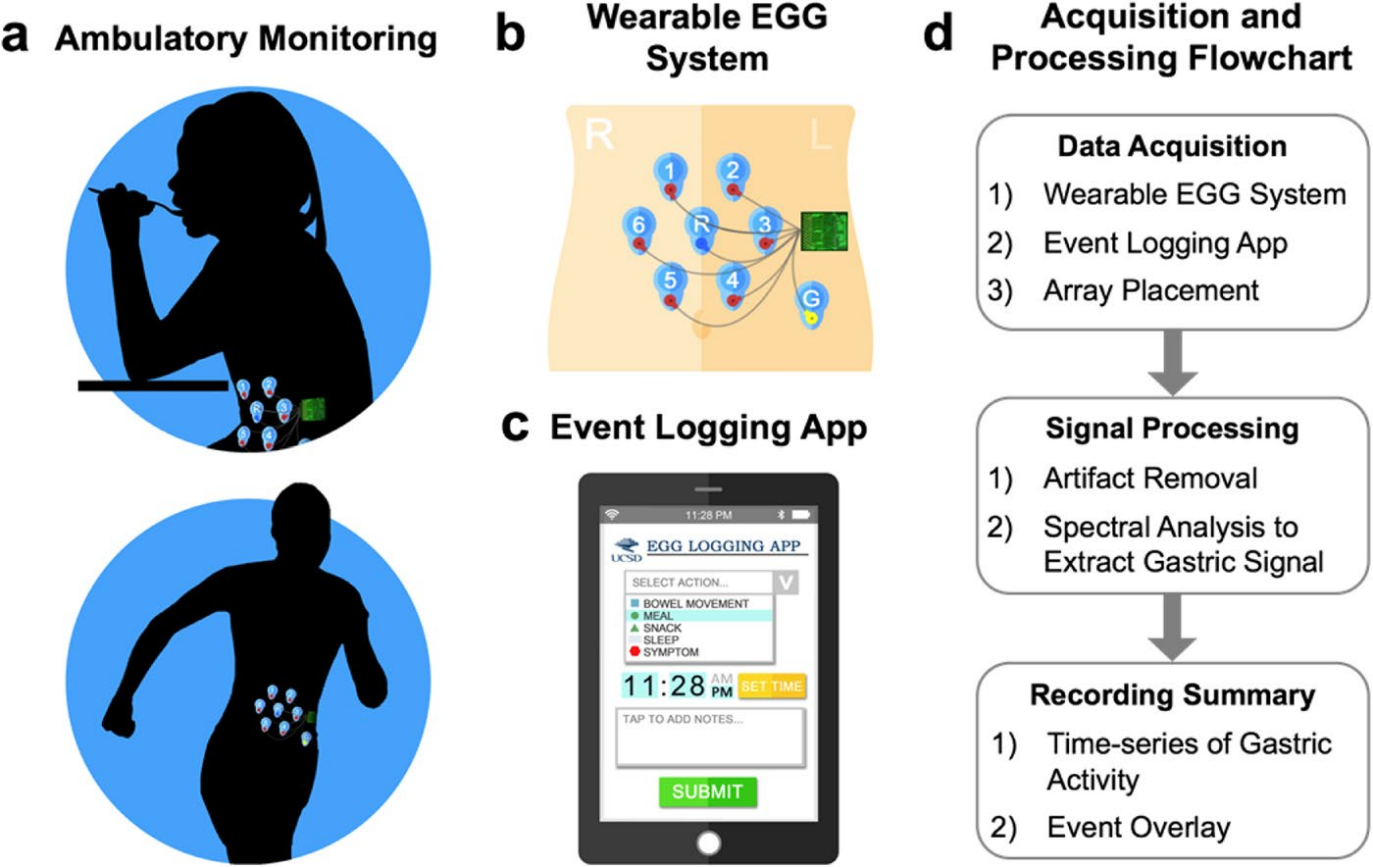
(**a**) Illustration of an ambulatory monitoring system to record gastric electrical activity during unrestricted living outside of the clinic, which includes (**b**) the recording hardware with skin-mounted electrodes, and (**c**) a smartphone application for logging events. (**d**) Workflow includes processing of recordings to remove artifacts, extract gastric activity, and synchronize with event timing such that clinical assessments can be made.

Here, we present a fully automated and computationally efficient statistical approach for removing artifacts from the EGG signal. In addition, we demonstrate a wearable EGG system for ambulatory monitoring with automated artifact removal algorithms, and a data logger with the ability for the user to tag events (e.g., symptoms, meals, etc.) of interest (see Fig. 1). We successfully validate the approach using simultaneous gastric manometry recordings, a clinical gold standard for GI motility assessment. We further demonstrate this system’s use in free-living subjects during 24-hour ambulatory monitoring, where we observe dynamics of gastric emptying spanning meals along with a daily gastric circadian oscillation.

## Results

### Effect of Artifact Rejection on EGG Waveforms

**Figure 2:**
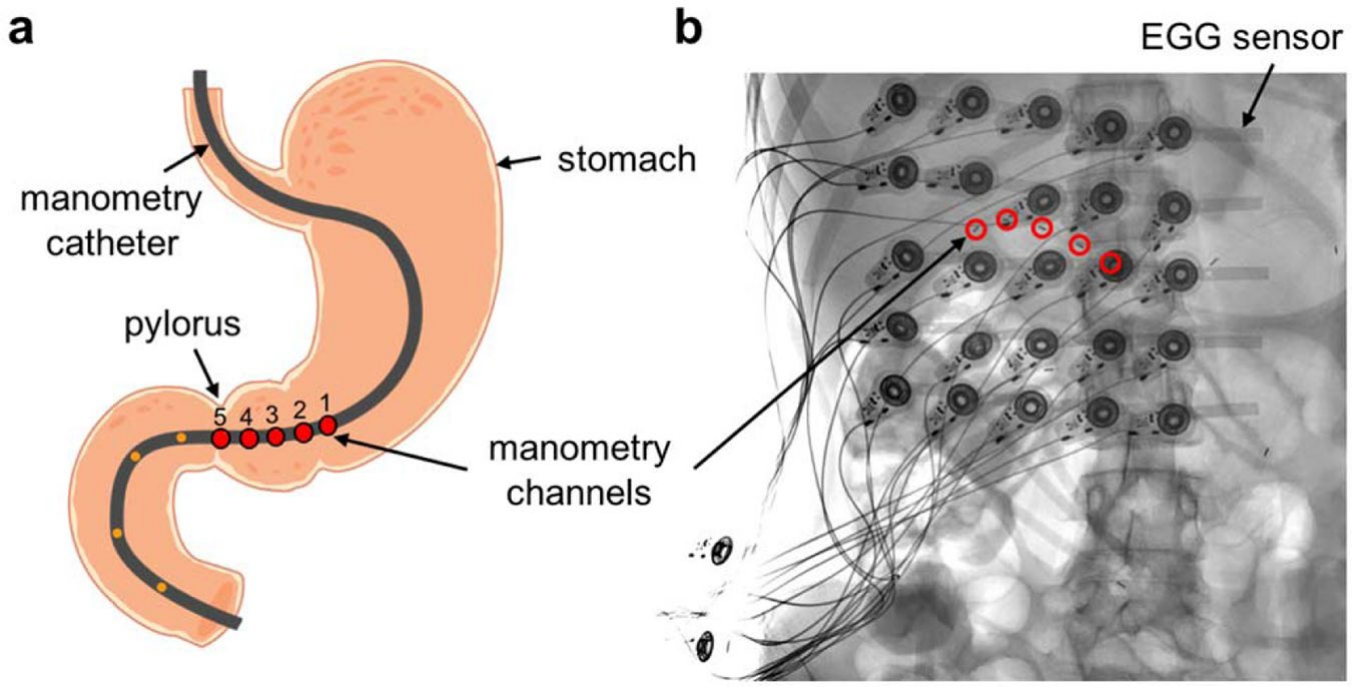
(**a**) Cartoon of the stomach depicting the placement of the manometry catheter. The red dots indicate the locations where the pressure is measured; five sensors in the antrum with 1 cm spacing. (**b**) An X-ray image of a subject showing the position of the EGG sensor array relative to the manometry channels.

We validated initial placement of electrodes for use in comparison to gastric manometry by direct visual mapping with the subject’s abdominal X-ray, whereby the two systems could be visualized simultaneously. Because we were uncertain of the contribution of electrode position to signal clarity, we initially applied a high-resolution 25 electrode grid across the front of the abdomen to ensure maximum capture of gastric signal. We confirmed that we were able to cover the stomach by using X-ray imagery to visualize the electrodes over the manometry pressure sensors inserted into each subject’s stomach (Fig. 2). All subjects stated that the system fit comfortably and was not irritating or limiting to motion.

**Figure 3:**
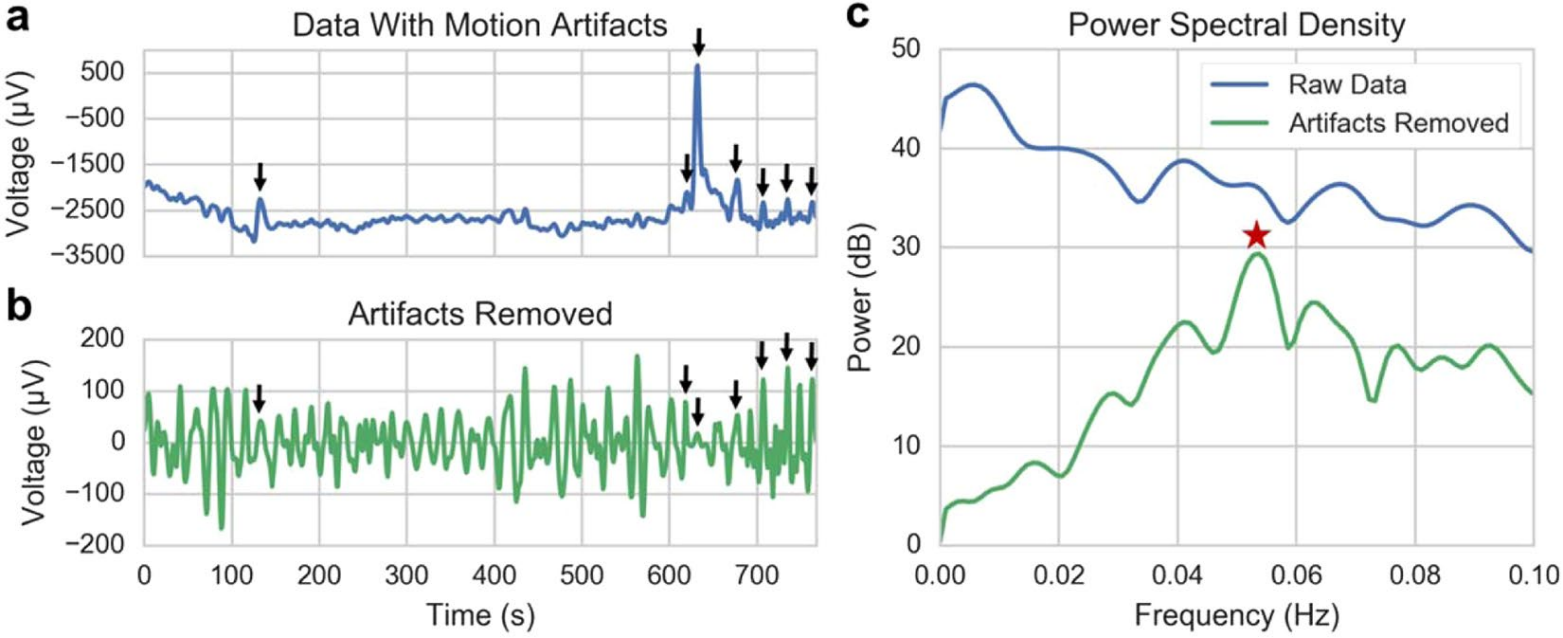
(**a**) Example time-series of raw EGG data containing motion artifacts (black arrows). (**b**) The same time-series following artifact removal using the LMMSE method (black arrows aligned by time with data in A). (**c**) A frequency domain representation of both time series (colors matched to a, b). The red star indicates the EGG frequency peak from this time series matching the expected frequency peak of gastric contractions, which only becomes apparent after removal of artifacts (green curve).

**Figure 4:**
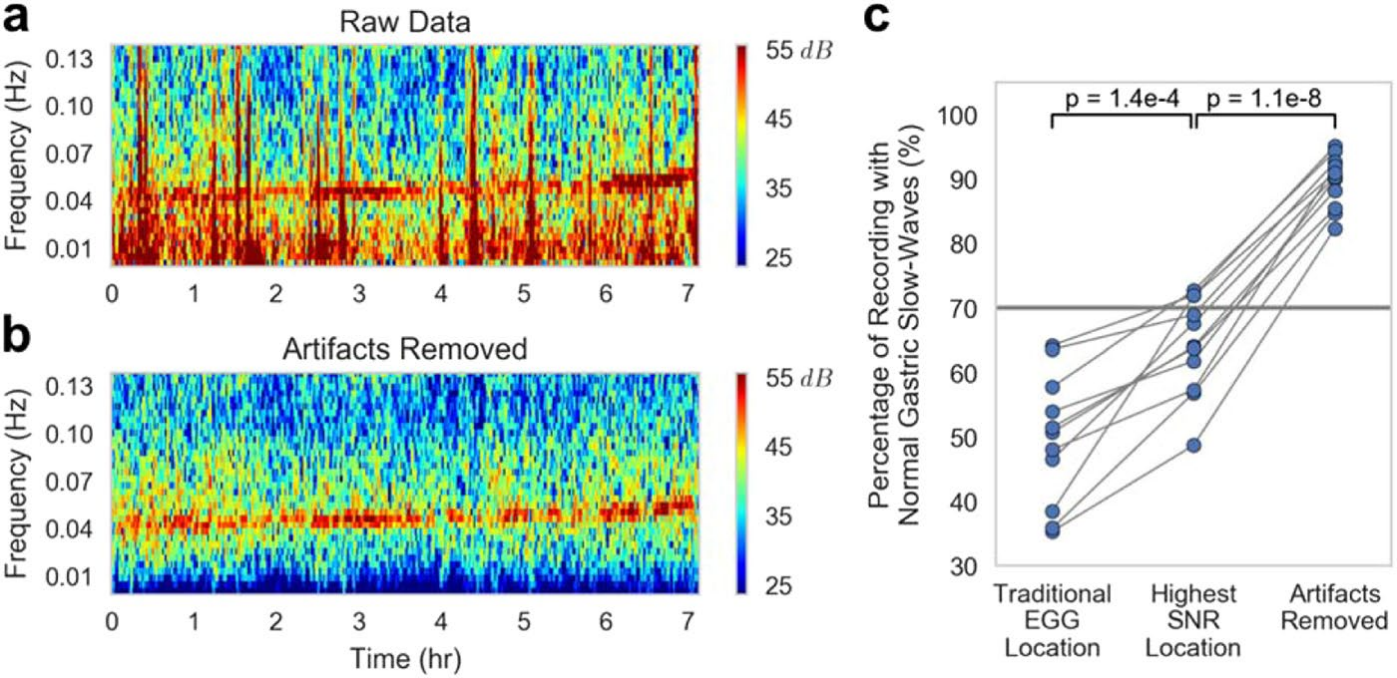
(**a**) Spectrogram of the raw EGG signal with motion artifacts visible as red vertical bands, and (**b**) spectrogram of the same signal with artifacts removed by the LMMSE method for Subject 2. (**c**) The percentage of normal gastric slow-wave activity across subjects using electrodes at the traditional EGG location (50 ± 10%), the highest SNR location (64 ± 8%), and the highest SNR location after artifact removal (90 ± 4%). Gastric activity is normal if the spectrum exhibits dominant power in the range of 2–4 cpm. In humans, the normal percentage of gastric slow-wave is typically defined as 70% (horizontal gray line).

The motion artifacts present in the EGG are typically manifested as short bursts (i.e., a few seconds or less) of high-amplitude activity (i.e., on the order of mV), compared to the slow rhythmic oscillation of the gastric activity at 0.05 Hz in the range of 50–200 μV. As such, instead of deleting windows of recordings with motion artifact, it is desirable to identify and suppress artifacts with appropriate estimation approaches (Fig. 3). The artifacts result in high broadband power that completely masks the EGG signal and does not allow for removal with band-pass filtering alone. Here, we used an interference cancellation approach where we first identified the artifact as interference in time using a linear minimum mean squared error estimation (LMMSE) with locally estimated mean and variance statistics (see Methods section for details), and subsequently subtracted it from the waveform. Implementing this procedure revealed a clear peak in the spectral representation of the resultant waveform around 0.05 Hz (Fig. 3c, red star), the peak expected from the EGG signal^34^. Spectrographic representations of the signal without artifact rejection (Fig. 4a) feature artifacts prominently, consistent with other findings from the literature^28^, and are prohibitively noisy without proper processing. After artifact removal, the 0.05 Hz gastric signal became clear (Fig. 4b), rendering further analysis feasible.

A commonly used feature in evaluating the EGG is the percentage of the recording with the maximum power between 2–4 cpm (i.e., the percentage of the recording with “normal” gastric slow-waves); below 70% is considered abnormal^34^. Traditionally, the EGG is recorded with a bipolar measurement from a pair of electrodes placed halfway between the xiphoid process and umbilicus, spaced 4–5 cm apart^22^. We found this configuration did not provide the highest EGG signal-to-noise ratio (SNR) across subjects. When comparing the traditional location to the highest SNR location from the electrode array (see Supplemental Table S1 for electrode locations), we found a significantly higher percentage of normal slow-waves in all 11 subjects (*p* = 1.4 × 10^−4^; Fig. 4c), which was further increased after artifact removal (*p* = 1.1 × 10^−8^; Fig. 4c). The combination of electrode placement and artifact removal resulted in the substantial increase in percentage of normal slow-waves from 50 ± 10% to 90 ± 4% (Table 1).

**Table 1:** Improvement in EGG percent 2–4 cpm activity and correlation coefficient between EGG power and manometry motility index with electrode placement and artifact removal (n = 11). Statistical significance after bonferroni correction for number of manometry channels.

Subject	Gender	Age (years)	Percent Normal Gastric Slow-Waves			EGG/Manometry Correlation (R-Value)			
			Traditional EGG Location	Highest SNR Location	Artifacts Removed	Traditional EGG Location	Highest SNR Location	Artifacts Removed	Manometry Channel
1	F	13	54	62	90	−0.18	0.48*	0.65*	#1
2	F	14	64	72	95	0.00	0.42*	0.74*	#3
3	M	7	51	64	88	−0.17	0.32*	0.33*	#4
4	F	15	35	49	82	−0.17	0.20*	0.50*	#2
5	F	12	36	57	85	0.17	0.40*	0.44*	#1
6	F	8	58	73	94	−0.13	0.21*	0.34*	#5
7	F	14	47	68	90	−0.26	0.02	0.58*	#5
8	M	10	64	69	93	−0.19	0.45*	0.46*	#2
9	M	15	48	57	92	0.28*	0.29*	0.57*	#5
10	F	15	38	72	91	0.21*	0.81*	0.82*	#5
11	F	17	51	64	85	0.58*	0.82*	0.82*	#5
Mean		**12 ± 3**	**50 ± 10**	**64 ± 8**	**90 ± 4**	**0.01 ± 0.26**	**0.40 ± 0.24**	**0.57 ± 0.17**	

*Correlation p-value < 0.01.

### Validation of EGG in Comparison to Gastric Manometry

**Figure 5:**
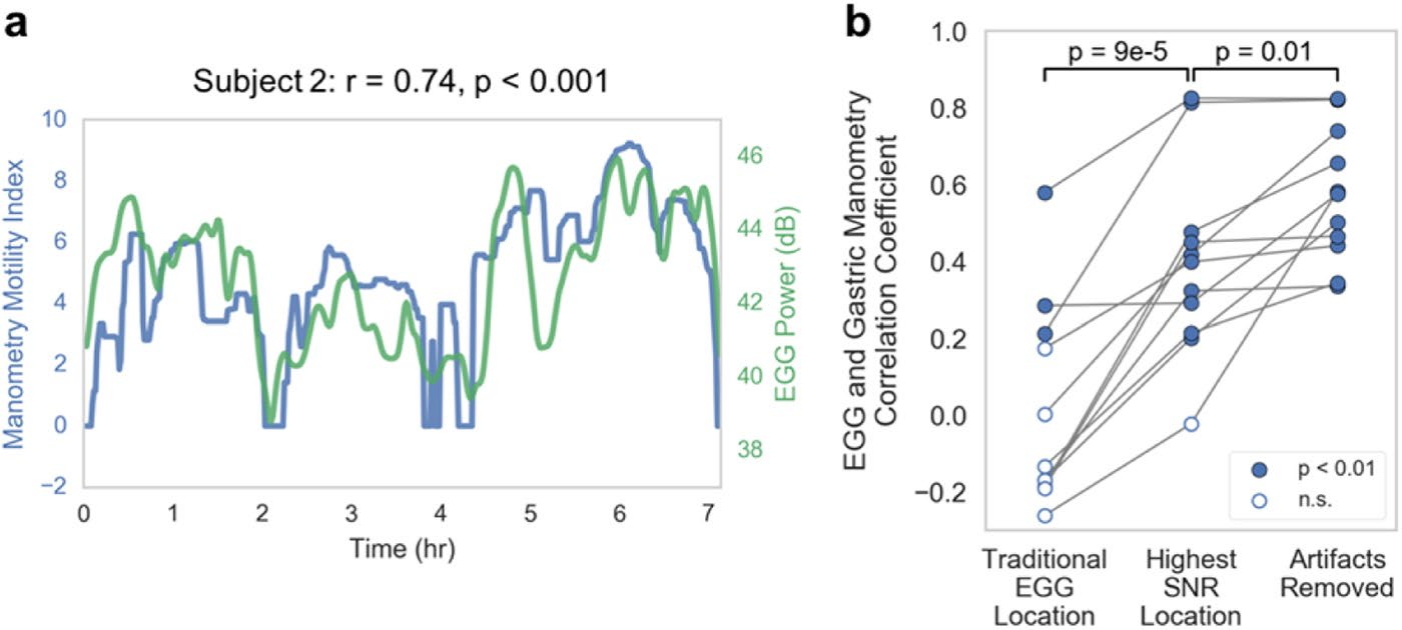
(**a**) Overlay of the EGG power between 0.04–0.06 Hz and the manometry motility index (r = 0.74, p < 0.001). (**b**) Correlation between EGG and manometry motility index across subjects using electrodes at the traditional EGG location (r = 0.01 ± 0.26), the highest SNR location (r = 0.40 ± 0.24), and the highest SNR location after artifact removal (r = 0.57 ± 0.17). Hollow circles indicate correlations that are not statistically significant positive correlations.

The power of the signal in the 0.04–0.06 Hz frequency band is representative of the magnitude of the gastric electrical activity^34^, and the manometry motility index is a measure of the contractility of the stomach measured from the invasive catheter. In 11 subjects, both invasive manometry pressure and noninvasive EGG were recorded simultaneously. A least-squares regression was computed between the mean EGG power and the manometry motility index for each channel to evaluate the relationship between the two recordings. Using electrodes at the traditional EGG location, we found a significant correlation between the two measures in only three of the subjects (mean r = 0.01 ± 0.26; Table 1). The agreement between manometry motility index and gastric electrical activity improved significantly in all subjects (*p* = 9 × 10^−5^) when using the EGG signal from the highest SNR location (mean r = 0.40 ± 0.24; Fig. 5b; Table 1). Artifact removal further increased correlation strength in every subject, causing a significant group improvement (mean r = 0.57 ± 0.17; *p* = 0.01; Fig. 5b; Table 1; for comparison of each individual manometry channel, see Supplemental Table S2). This improvement with electrode placement and artifact removal suggests the enhanced 0.05 Hz signal indeed corresponds to gastric activity.

### Ambulatory EGG Recordings

Based on our findings during validation against manometry, we were able to decrease the number of electrodes to include only those most likely to cover the stomach during ambulatory experiments and confirmed placement using ultrasound (Fig. 1b). This decrease provided improved ease of wearability over 24 hours. We, in parallel, developed an event-logging application that can be accessed with an internet-accessible mobile device to timestamp contextual events of interest (e.g., meals, snacks, etc) (Fig. 1c). Having confirmed that the platform and associated data collection approaches did indeed provide us with a functional system, we proceeded with experimental exploration of EGG using a data processing pipeline (Fig. 1d) we established for our experiments.

**Figure 6:**
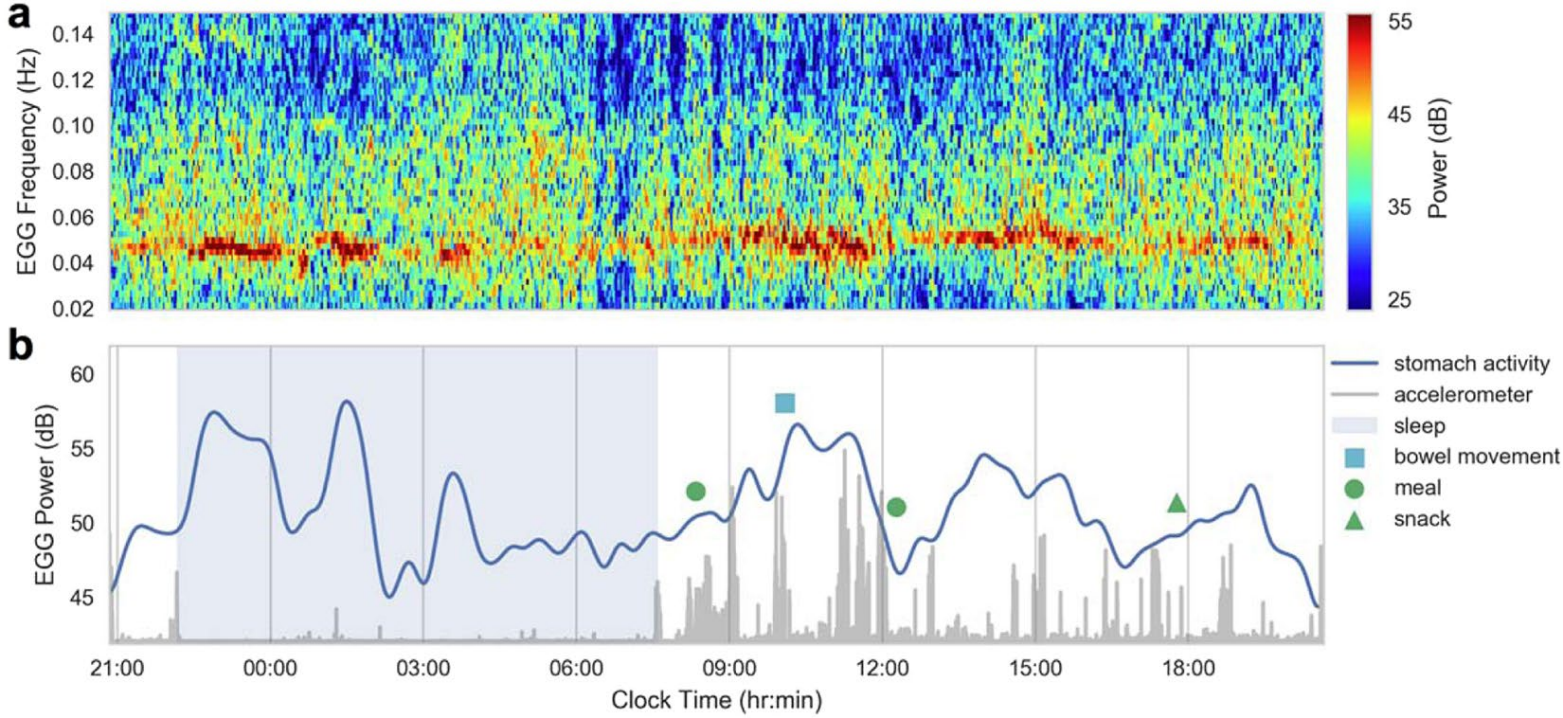
(**a**) Spectrogram representation of EGG over a 24 hour period after the removal of artifacts. The EGG signal can be seen in the 0.04–0.06 Hz frequency band. (**b**) Extracted EGG power (mean of the 0.04–0.06 Hz frequency band) with event markers. The accelerometer magnitude is plotted in gray.

**Figure 7:**
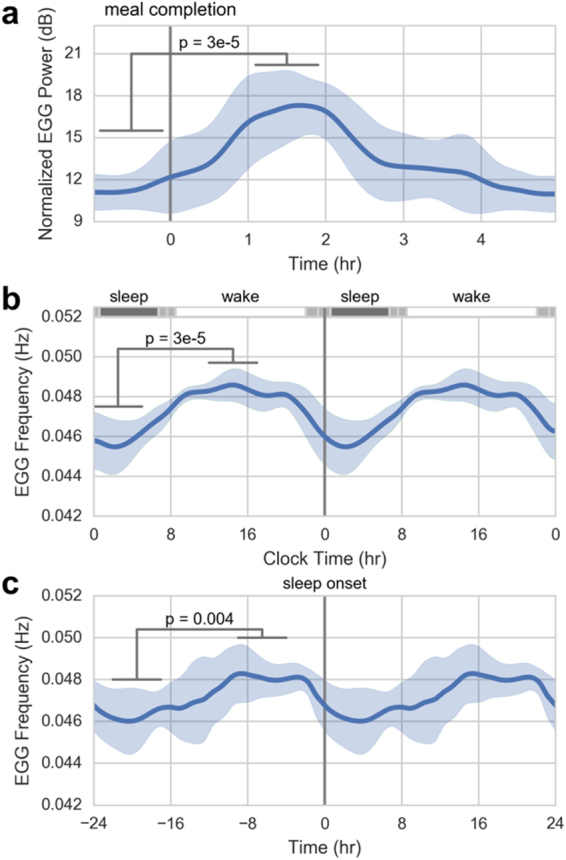
Ambulatory EGG extracted from multiple recordings (n = 8) on a single healthy subject. (**a**) Pre- and post-prandial EGG power response to isolated meals (n = 6). The time of meal completion is indicated by the vertical gray line. (**b**) High resolution mean of the EGG frequency from multiple continuous recordings from a single subject throughout a day (n = 8). Average time with the subject asleep in dark gray at top, +/− one standard deviation (light gray), with average time in wake in white. (**c**) The same signals averaged by time of sleep onset rather than by time of day. Differences in shape and distribution of variance suggest unique contributions of circadian and sleep regulation to GI activity profiles. The error bars are +/− one standard deviation.

The automated method for suppressing artifacts enables the robust recording of gastric electrical activity in an ambulatory setting with a wearable EGG system (see Supplemental Fig. S1 for an example of artifacts observed in ambulatory recordings). We used such a system to record continuously for eight separate 24-hour sessions in a free-living subject (see Fig. 6 for data from a representative day). We found substantial change in power across the day with an apparent signature around meal times, which we then sought to substantiate. To quantify changes in gastric activity before and after meal consumption during the 24-hour sessions, we assessed isolated meals (n = 6), defined as meals that occurred after at least five hours of fasting along with no other reported events for five hours after the meal. The EGG power, normalized to background noise, showed a significant effect of time-from-meal-completion (Fig. 7a, vertical gray line) (*p* = 3 × 10^−5^). The mean normalized power increased from 11.3 dB to 17.0 dB after the meal, and the pattern took 3–4 hours from meal completion to return to baseline, consistent with clinically observed gastric emptying^35,36^.

Separate from the effects of meals on EGG power, we also observed a significant change in EGG dominant frequency across the day. We identified the dominant frequency of the EGG throughout the 24 hours and averaged the eight recordings. We found a significant effect of time on EGG dominant frequency when recordings were aligned by clock time (*p* = 3 × 10^−5^, Fig. 7b). The mean frequency was 2.74 cpm (0.0457 Hz) at night and increased to 2.91 cpm (0.485 Hz) during the day. The moment of lowest across-recording variance was in the morning rise, suggesting that this is the most conserved feature when recordings are aligned by time of day. We also found a significant effect of time on EGG frequency when recordings were aligned by time-from-sleep-onset (*p* = 0.004, Fig. 7c); moreover, this pattern of daily frequency modulation was distinct from that seen in alignment by time of day. Specifically, the point of the lowest variance was immediately before sleep onset.

## Discussion

We have demonstrated a new ambulatory recording system and analysis methodology that allows for continuous and noninvasive measurements of stomach electric activity in unrestricted individuals across 24 hours. By identifying and removing artifacts rather than deleting time windows in which they occur, we were able to increase the accuracy with which EGG signals can be used to reconstruct internal contractions, as validated with gastric manometry. This system allowed us to measure stomach activity in response to a meal, revealing a time course of dynamics that matches clinically observed stomach emptying times. It has not previously been possible to observe such high-resolution meal-related GI dynamics by noninvasive means in free-living subjects. Because of the rich and unexplored feature space that these continuous EGG recordings generate, continuous monitoring of patients’ GI activity now has the potential to enable identification of personal profiles of EGG change that help describe idiopathic pathologies, optimize medical treatments, and evaluate effects of meal composition, exercise, etc., on gastric health. To further explore this possibility, we assessed EGG across 24 hours, and found modulation of dominant frequency across the day. Interestingly, we found distinct contributions from sleep and daily timing, suggesting differing contributions from homeostatic and circadian regulators. The work presented here provides a way to measure these relationships in real-life settings with healthy and clinical populations, such that healthy dynamics can be described, and eventually, unhealthy dynamics may be predicted, treated, or prevented.

Our observation of a postprandial pattern of gastric activity lasting 3–4 hours was consistent with typical gastric emptying times^35,36^. Gastric emptying is the process by which the stomach empties itself of food content following a meal. Gastric emptying studies have observed high inter- and intra-subject variability^37–39^, but repeated studies on patients are usually avoided to limit radiation exposure from the radioactive tracer required^40^. Given the importance of circadian rhythms, sleep, and meal timing in GI and metabolic health^41–45^, our ambulatory system enables studies of multiple meals across the daily wake/sleep cycles, without loss of signal over time as with radioactive dyes, and without needing to administer repeated doses of marker to cover longer time spans of observation in a laboratory or clinical setting. Further validation of our results, by comparison to gastric scintigraphy and during polysomnography studies for sleep contributions, over a large and demographically diverse population, may further elucidate which specific patterns are informative for which individuals.

While the underlying bioinstrumentation acquisition system was modified from existing ECG standards, a number of unique problems presented themselves in developing methods to analyze the resulting EGG waveforms. Unlike the ECG, time-domain EGG waveforms are difficult to interpret, as the signals do not have a signature morphology like the heart’s QRS complex. Because of the 0.05 Hz rhythmic gastric slow-wave, spectral analysis is of particular importance for analyzing EGG data, with large windows of data required for adequate frequency resolution of the low frequency signal^46^. Spectral analysis provides changes in gastric power and frequency, but short-duration large-amplitude artifacts still pose spectral analysis limitations, prior to the time-domain artifact rejection paradigm we present here. There are several advantages to the approach described in this manuscript for removing artifacts from the EGG signal. Small segments of the time series are processed independently, enabling efficient computation and scalable parallel signal processing, which is useful for real-time applications such as biofeedback^47,48^. Unlike supervised machine learning approaches^49^, this method does not require human-labeled training data. Unlike independent component analysis^30^, this method can remove non-stereotyped artifacts from the signal, which are the most common type of artifact in EGG recordings. Finally, segments of the time-series with artifacts are not eliminated, which results in more accurate spectral decompositions within the window and preserves the time of day necessary to associate with logged events. It should be noted that this method would not allow for complete recovery of the EGG signal during sustained artifacts, such as during vigorous exercise.

To test the accuracy of our methodology, we considered the clinical “gold-standard” for motility measurements, gastric manometry, since there is no commercially available technology for simultaneous serosal or mucosal measurements of gastric electrical activity in awake subjects to use as comparison. We expected an association between manometry and the EGG, since smooth muscle contractions in the stomach are generated by electrical activity. An increase in EGG amplitude has been shown to be associated with stronger contractions of the stomach^50,51^ and not simply from the full stomach moving closer to the electrodes^52^. However, we did not expect a perfect correlation between gastric manometry and EGG due to several limitations besides inherent measurement noise. The manometry catheter has five closely spaced sensors that are positioned in the antrum of the stomach, but it can migrate during the study from peristaltic contractions^53^ and from gastric accommodation due to a meal^54^ requiring repositioning. Also, manometry can only detect lumen occluded contractions^55,56^; more sensitive motility measures have shown a better agreement with the gastric slow-wave^57^. Finally, slow-waves from the corpus of the stomach may be contributing to the EGG signal, which the manometry did not measure. Nonetheless, we found that using multiple electrodes, along with our artifact rejection methodology, produced a dramatic improvement in the correlation between the two modalities across all subjects. Future studies with more sensitive measures of gastric motility or electrical activity are necessary to further validate multichannel EGG findings.

A pediatric population was used in this study because Rady Children’s Hospital is the only location in San Diego county that performs gastric manometry. Motion artifacts appear indistinguishable in pediatric and adult recordings since the underlying mechanism that generates artifacts (i.e., movement and skeletal muscle contractions) are the same, nor do the age of the subjects alter our application of the noise removal techniques described here. The lack of availability to perform adult gastric manometry in San Diego county illustrates the limited scope of application of this procedure as well as the need by both researchers and clinicians for easier-to-apply alternatives; the demonstrated approach is perhaps a step in that direction.

While the subjects in this study all had normal 2–4 cpm gastric activity throughout the recording, patients with gastric disorders typically exhibit desynchronized activity, including tachygastria (above 4 cpm) and bradygastria (below 2 cpm)^21,22^. The approach described in this manuscript removes baseline drift and short bursts of high-amplitude activity due to motion artifacts, while preserving the frequency bands that contain normal gastric activity and tachygastria (see Supplemental Fig. S1). On the other hand, detection of bradygastria may be more challenging, because significant baseline drift is prone to occur during ambulatory recordings. Reducing motion-related drift can possibly be achieved in the future with active electrodes^58^, novel dry electrode designs^59^, or both. Also, in this study, we performed spectral analysis with parameters traditionally used in the EGG literature to isolate the performance of our artifact removal methodology. Four-minute windows of data provide high frequency resolution around 0.05 Hz, albeit at the cost of temporal resolution. Future work can utilize modern efficient statistical signal processing methods with improved spectrotemporal resolution, such as multi-taper methods^60^, or Bayesian state-space methods that can exploit dynamic group sparsity^61^.

Future iterations of this ambulatory recording system should become smaller and less noticeable to the subject to facilitate continuous use^62^. Recent developments of flexible and stretchable skin-mounted electronics^63^, as well as innovations in microfabrication procedures necessary to produce such systems at scale^64,65^ will enable improved wearability without sacrificing fidelity. In considering how to refine the wearable system and reduce obtrusiveness, it is important to recognize that there are advantages to using multiple measurement electrodes. Specifically, we found the EGG signal to be sensitive to electrode placement in this study. Selecting the measurement electrodes with the highest EGG SNR resulted in significant increases in both the percentage of normal gastric slow-waves and the correlation with manometry. Since we expect a decrease in signal amplitude with increasing distance between the stomach and electrodes^66^, this demonstrates that gastric anatomical variability between subjects^67^ is considerable enough to have a significant effect on the EGG signal. Additional measurement electrodes make it more likely that the stomach will be captured under the recording surface, obviating the need to use imaging to guide placement. An additional benefit of using electrode arrays is the ability to estimate motility propagation parameters, as we have recently shown^68^. Currently, there is no established standard electrode placement for the EGG, so balancing greater coverage with device miniaturization is an active area of research. It should be noted that high BMI individuals were not included in this study. Further investigations with volumetric imaging or modeling are necessary to assess the effect of subcutaneous adipose tissue on the EGG.

There are many advantages and opportunities that noninvasive ambulatory monitoring of gastrointestinal function provides. Recording for longer periods increases the likelihood of capturing abnormal myoelectrical events^62^. The ambulatory EGG monitoring is a less expensive alternative to in-clinic monitoring, which involves use of clinical staff and facilities. Children can be monitored at home, which reduces stress on both children and their parents. Since it is noninvasive, repeated recordings can be easily performed to objectively guide treatment. Wearable monitoring systems can be disseminated to patients living in distant locations, allowing for remote assessment and treatment of patients. For example, capturing many meals and symptomatic events enables assessment of intrasubject variability between days, meal composition, etc. This approach can allow the generation of large, longitudinal datasets on which machine learning may find predictive patterns not visible to human clinicians, as has happened in other areas of healthcare^69,70^. Finally, the prolonged monitoring allows for research on the implications of circadian and sleep disruptions on GI disorders^41–43^. Wearable ECG technology has generated substantial gains in detection of events, enabling faster and more accurate diagnoses and interventions^62,71,72^. A similar transformation for patient care should be possible for GI and metabolic disorders through refinement of wearable EGG systems.

## Methods

### Artifact Rejection Methodology

Since the artifacts typically have much higher amplitude compared to the EGG (50–200 μV), we can estimate the artifacts in the signal, and subsequently subtract them from the observed data to extract only the EGG component in the signal. In our recordings, the EGG signal, artifact, and noise characteristics are non-stationary (i.e., they vary significantly throughout the recording). To accurately estimate the artifacts, we can adapt the processing to the local characteristics of the data by using relevant information in the neighborhood region centered around that time point. A similar approach has been previously proposed for image processing, where the filter is adapted at each pixel^73^.

We formulate the problem as follows:

1 $$y=x+e$$

where *y* is the observed signal, *x* is the artifact, and *e* is the EGG signal. By choosing a window size of *n* to be the average EGG cycle duration (i.e., inverse of the mean peak EGG frequency), we can assume the following:

2 $${\boldsymbol{{\rm E}}}[e]=0,\,{\rm{Var}}(e)={\sigma }_{e}^{2}$$

Under this assumption, a filtering algorithm was obtained using the linear minimum mean-squared error estimator (LMMSE):

3 $$\hat{x}={\boldsymbol{{\rm E}}}[y]+\frac{{\rm{Var}}(y)-{\sigma }_{e}^{2}}{{\rm{Var}}(y)}(y-{\boldsymbol{{\rm E}}}[y])$$

where the expectation and variance of the signal *y* are given by the local mean and variance. Since the variance of the observation is the sum of the variances of the artifact and the EGG, both non-negative, the variance of the recorded signal should be greater than or equal to the EGG variance. In certain windows of the real data, the local variance can have a calculated value less than the EGG variance. When this happens, the local variance is set to ${\sigma }_{e}^{2}$. Since the value of ${\sigma }_{e}^{2}$ is not exactly known, a slight variant of the LMMSE is used:

4 $$\hat{x}={\boldsymbol{{\rm E}}}[y]+\frac{{\rm{\max }}\{0,\,{\rm{Var}}(y)-{\sigma }_{e}^{2}\}}{{\rm{\max }}\{{\rm{Var}}(y),{\sigma }_{e}^{2}\}}(y-{\boldsymbol{{\rm E}}}[y])$$

where ${\sigma }_{e}^{2}$ is calculated by taking the mean of all values of the local variance of *y* over the time series. In practice, this LMMSE has the effect of “filtering” out the waveform in regions without artifact and leaves the data unchanged in the vicinity of artifacts. The resultant “artifact signal” is then simply removed from the raw data as follows:

5 $$e=y-\hat{x}$$

For a more complete mathematical derivation, see Supplemental Materials.

### Simultaneous Manometry and EGG Recordings

We used manometry to record GI contractions in 11 human subjects (age: 7–17 years, gender: 3 M/8 F, BMI: 19 ± 3). In children, dyspepsia symptoms are more common in girls^74^, so recruitment for subjects was biased toward greater inclusion of female subjects of this age group. All subjects had a healthy enteric nervous system, characterized by having at least one Phase III migrating motor complex during fasting. We performed manometry with a flexible catheter consisting of eight water-perfused channels, and monitored pressure at either four or five channels (1 cm spacing) positioned in the antrum of the stomach (see Fig. 2A). The recording duration was between six to eight hours, with at least four hours of fasting followed by two hours after a meal. We simultaneously recorded multichannel EGG with a five-by-five array (2 cm center-to-center spacing) of skin-mounted electrodes positioned over the stomach during the manometry study^68^, with ground and reference electrode placed on the subject’s left side below the rib cage. We used the radio-opaque markers on the manometry catheter at the pressure measurement sites to confirm appropriate placement of the EGG electrode array (see Fig. 2B). The subjects’ skin was gently abraded with Nuprep® gel prior to electrode placement to lower the skin impedance and minimize motion artifacts.

We recorded the EGG at a sampling rate of 250 Hz and downsampled to 5 Hz prior to artifact removal and analysis to decrease computational time. Although one of the electrodes was chosen as the reference during the recording, the channels can be re-referenced after the recording to any of the other channels by simple subtraction of the time-series. For example, to compute the traditional EGG signal, we re-referenced the data to the electrode on the midline halfway between the xiphoid process and umbilicus and used a measurement electrode 4 cm to the subject’s left^22^. We computed a short-time Fourier transform spectrogram from every pair of measurement electrodes to determine the pair with the highest signal-to-noise ratio (SNR). To generate the spectrograms, we divided the time domain data into consecutive four-minute segments with 75% overlap and applied a Hamming window to each. We defined SNR as the average power (in dB) between 0.04 Hz and 0.06 Hz subtracted from the average power in all other frequencies between 0.02 Hz and 0.20 Hz. We calculated motility index, a measure of contractility, from the manometry recording using four-minute windows with 75% overlap to match the EGG spectral analysis. We defined motility index as the natural logarithm of the area under manometric pressure peaks above a threshold pressure of 9 mm Hg^75^. We evaluated the correlation between the mean EGG power from the 0.04 Hz to 0.06 Hz frequency band and manometry motility index using linear least-squares regression analysis.

### Ambulatory EGG Recordings

We developed a system capable of robustly recording the EGG activity in an ambulatory (i.e., free-living, unrestricted) setting. Figure 1 illustrates the components of the system, which includes the hardware, electrodes, and smartphone application. The hardware consists of a low noise, eight channel biopotential amplifier with a 24-bit analog to digital converter designed for EEG applications (Texas Instruments ADS1299), microSD card slot for local storage of data for offline analysis, and an accelerometer for tracking subject movement. The battery and microSD card capacities were chosen to satisfy the duration requirements of the recording. In our studies, an 8GB microSD card and 3.7 V 1800mAh battery provided sufficient memory and power to allow for 24-hour recordings. We used the shortest possible electrode cable length (10 cm) to limit motion artifacts from cable movement^76^. Off-the-shelf Ag-AgCl electrodes typically used for long-term monitoring in cardiology were used for ambulatory EGG recordings. We developed a smartphone application to enable the subject to document events or activities that are time-synchronized to the EGG recording. Examples include meals, snacks, bowel movements, sleep and wake onset, symptoms, etc.

We conducted eight 24-hour ambulatory EGG recordings on a single healthy adult subject (male, age = 28, BMI = 22.6) over the course of a 6-month period. We performed an ultrasound scan to locate the stomach after the subject consumed of 500 mL of water. We arranged six measurement electrodes in a radial array (3 cm inter-electrode spacing) with the reference electrode in the middle of the array and the ground on the subject’s left side (see Fig. 1b). The reference electrode was positioned 5 cm below the xiphoid process. The subject was instructed to conduct routine daily activities and refrain from showering, while logging meals, exercise, bowel movements, and sleep using the smartphone application.

Processing of the ambulatory EGG data was the same as described above for the simultaneous manometry and EGG recordings. To assess the change in gastric myoelectric activity due to consumption of a meal, we averaged the normalized EGG power for six isolated meals starting from one hour prior to meal completion to five hours after (Fig. 7a). We defined the normalized EGG power as the mean power in the 0.04–0.06 Hz band minus the background noise level in the 0.06–0.10 Hz band to control for noise variability between recordings and channels. We extracted the dominant EGG frequency across time for each recording, defined as the maximum frequency in each four-minute window between 0.02 and 0.20 Hz, and plotted the averages across days to evaluate circadian effects (Fig. 7b). We aligned the data by sleep onset rather than clock time to determine if there were also separate sleep contributions (Fig. 7c).

### Statistics

We used a paired, two-sided t-test for the null hypothesis that two related samples have identical expected values to evaluate statistical significance when comparing the effect of artifact removal on percentage of normal gastric slow-waves and the correlation between EGG and manometry motility index (Figs 4c and 5c). To evaluate the statistical significance of the correlation between EGG and manometry motility index for each subject, we calculated a two-sided p-value for the hypothesis test whose null hypothesis is that the slope of the linear least-squares regression for two sets of measurements is zero (Table 1). To account for repeated measures, we applied Bonferroni correction of the p-values based on the number of manometry channels. We used an unpaired, two-sided test for the null hypothesis that two independent samples have identical expected values, assuming equal variances to evaluate the statistical significance of the effect of the meal on EGG power (Fig. 7a), effect of time of day on EGG frequency (Fig. 7b), and the effect of sleep on EGG frequency (Fig. 7c). We considered p-values of 0.01 or less to be significant.

### Ethics Statement

All human subject research was carried out at the University of California, San Diego and Rady Children’s Hospital, San Diego, and all study protocols and consent documents were approved by the institutional review boards at both institutions. We obtained informed consent from all subjects who participated in this study (including subject’s guardians for minors) and methods were performed in accordance with relevant regulations and guidelines.

### Data availability

Data were analyzed using in house code developed in Python v3.5.2 using Jupyter Notebook with the following modules: Numpy v1.11.2, Scipy v0.18.1, Pandas v0.18.1, Matplotlib v1.5.3 and Seaborn v0.7.1. All figures were made in Python v3.5.2 and formatted using Adobe Photoshop CC. The data generated in this work will be made available from the authors upon request.

## Electronic supplementary material

Supplementary Materials
