# Supplementary material for: Artifact Rejection Methodology Enables Continuous, Noninvasive Measurement of Gastric Myoelectric Activity in Ambulatory Subjects
Source: Sci Rep. 2018 Mar 22;8:5019. doi: 10.1038/s41598-018-23302-9 (PMC5864836; doi:10.1038/s41598-018-23302-9)
Supplement: Supplementary file 1 — Supplementary Materials [file 41598_2018_23302_MOESM1_ESM.pdf]

Artifact Rejection Methodology Enables Continuous, Noninvasive Measurement of Gastric Myoelectric Activity in Ambulatory Subjects

Authors:

Armen A. Gharibans [1,2], Benjamin L. Smarr [3], David C. Kunkel [4], Lance J. Kriegsfeld [3], Hayat M. Mousa [2,5], Todd P. Coleman [1,*]

Affiliations:

1. Department of Bioengineering, University of California at San Diego, La Jolla, CA, United States
2. Department of Pediatrics, University of California at San Diego, La Jolla, CA, United States
3. Department of Psychology, University of California at Berkeley, Berkeley, CA, United States
4. GI Motility & Physiology Program, University of California at San Diego, La Jolla, CA, United States
5. Neurogastroenterology and Motility Center, Rady Children's Hospital at San Diego, CA, United States

*Corresponding Author:

Todd P. Coleman
tpcoleman@ucsd.edu
(617) 308-8633
9500 Gilman Drive - PFBH 251
La Jolla, CA 92093-0412

Supplemental Materials

Derivation of the LMMSE to Remove Artifacts from the EGG

We can formulate the problem as follows:

$$y = x + e \tag{S.1}$$

where, y is the observed signal, x is the artifact, and e is the EGG signal. Within a window of size n samples the local mean and variance of y can be computed:

$$\mathbf{E}[y]_i = \frac{1}{2n+1}\sum_{k=i-n}^{n+i} y_k \tag{S.2}$$

$$\mathrm{Var}(y)_i = \frac{1}{2n+1}\sum_{k=i-n}^{n+i} \left(y_k - \mathbf{E}[y]_i\right)^2 \tag{S.3}$$

By choosing n to be the average EGG cycle duration (i.e., inverse of the mean peak EGG frequency), we can assume the following:

$$\mathbf{E}[e] = 0 \tag{S.4}$$

$$\mathrm{Var}(e) = \sigma_e^2 \tag{S.5}$$

Now, we can apply the LMMSE:

$$\hat{x} = \mathbf{E}[x] + \left(\frac{\mathrm{Cov}(x,y)}{\mathrm{Var}(y)}\right)(y - \mathbf{E}[y]) \tag{S.6}$$

For our problem,

$$\mathbf{E}[y] = \mathbf{E}[x] \tag{S.7}$$

$$\mathrm{Cov}(x,y) = \mathrm{Cov}(x+e) = \mathrm{Cov}(x,x) + \mathrm{Cov}(x,e) = \mathrm{Var}(x) \tag{S.8}$$

Therefore, the LMMSE for the artifacts in our signal becomes:

$$\hat{x} = \mathbf{E}[x] + \frac{\mathrm{Cov}(x,y)}{\mathrm{Var}(y)}(y - \mathbf{E}[y]) \tag{S.9}$$

$$= \mathbf{E}[y] + \frac{\mathrm{Var}(x)}{\mathrm{Var}(y)}(y - \mathbf{E}[y]) \tag{S.10}$$

$$= \mathbf{E}[y] + \frac{\mathrm{Var}(y) - \sigma_e^2}{\mathrm{Var}(y)}(y - \mathbf{E}[y]) \tag{S.11}$$

Because the variance of the observation is the sum of the variances of the artifact and the EGG (due to an assumption of statistical independence), both non-negative, the variance of the recorded signal should be greater than or equal to the EGG variance. In certain windows of the real data, the local variance can have a calculated value less than the EGG variance. When

this happens, the local variance is set to σ_e^2. In other words, the local variance is expressed more precisely as:

$$\mathrm{Var}(y) = \max\left\{\mathrm{Var}(y), \sigma_e^2\right\} \tag{S.12}$$

The value of σ_e^2 is not exactly known, so a slight variant of the LMMSE can be used:

$$\hat{x} = \mathbf{E}[y] + \frac{\max\{0, \mathrm{Var}(y) - \sigma_e^2\}}{\max\{\mathrm{Var}(y), \sigma_e^2\}} (y - \mathbf{E}[y]) \tag{S.13}$$

where σ_e^2 is calculated by taking the mean of all values of the local variance of y over the entire time series.

In practice, the LMMSE filters out EGG in regions without artifact and leaves the data unchanged in the vicinity of artifacts. The artifacts can then be simply removed from the raw data as follows:

$$e = y - \hat{x} \tag{S.14}$$

Supplemental Table S1. Locations of traditional EGG and highest EGG SNR bipolar electrode pairs in 25 electrode array (5x5, 2cm spacing). All positions are relative to a reference point (0, 0) halfway between xiphoid process and umbilicus.

Subject	Traditional EGG Electrode Location (cm)		Highest EGG SNR Electrode Location (cm)		Distance Between Traditional and Highest SNR Electrodes (cm)	
	A (x, y)	B (x, y)	A (x, y)	B (x, y)	A	B
1	(0, 0)	(4, 0)	(-4, 4)	(2, 2)	5.7	2.8
2	(0, 0)	(4, 0)	(4, 2)	(-2, 0)	4.5	6.0
3	(0, 0)	(4, 0)	(-2, -2)	(-2, -4)	2.8	7.2
4	(0, 0)	(4, 0)	(-4, 4)	(4, 2)	5.7	2.0
5	(0, 0)	(4, 0)	(2, 4)	(2, -2)	4.5	2.8
6	(0, 0)	(4, 0)	(-4, 2)	(2, 2)	4.5	2.8
7	(0, 0)	(4, 0)	(0, 6)	(-4, 0)	6.0	8.0
8	(0, 0)	(4, 0)	(2, 4)	(-2, 0)	4.5	6.0
9	(0, 0)	(4, 0)	(0, 0)	(0, 6)	0.0	7.2
10	(0, 0)	(4, 0)	(0, 4)	(6, -4)	4.0	4.5
11	(0, 0)	(4, 0)	(-4, 0)	(4, 0)	4.0	0.0
Mean					4.2 ± 1.7	4.5 ± 2.6

Supplemental Table S2. Correlation of EGG with manometry motility index from each channel using highest SNR EGG electrodes and after artifact removal.

Subject	Antrum #1	Antrum #2	Antrum #3	Antrum #4	Antrum #5	Max	Mean
1	0.65	0.64	0.55	0.46	0.43	0.65	0.55 ± 0.10
2	0.22	0.57	0.74	0.74	0.54	0.74	0.56 ± 0.21
3	0.19	0.31	0.33	0.25	0.27	0.33	0.27 ± 0.05
4	n/a	0.50	0.00	0.02	0.37	0.50	0.22 ± 0.25
5	0.44	0.36	0.30	0.27	0.20	0.44	0.31 ± 0.09
6	-0.13	-0.22	-0.01	0.25	0.34	0.34	0.05 ± 0.24
7	-0.11	-0.10	-0.12	0.19	0.58	0.58	0.09 ± 0.30
8	0.34	0.46	0.39	0.42	0.21	0.46	0.36 ± 0.10
9	0.27	0.02	0.27	0.30	0.57	0.57	0.29 ± 0.20
10	-0.42	-0.14	0.18	0.71	0.82	0.82	0.23 ± 0.53
11	n/a	0.02	0.34	0.68	0.82	0.82	0.47 ± 0.36
Mean	0.16 ± 0.33	0.22 ± 0.31	0.27 ± 0.25	0.39 ± 0.24	0.47 ± 0.22	0.57 ± 0.17	

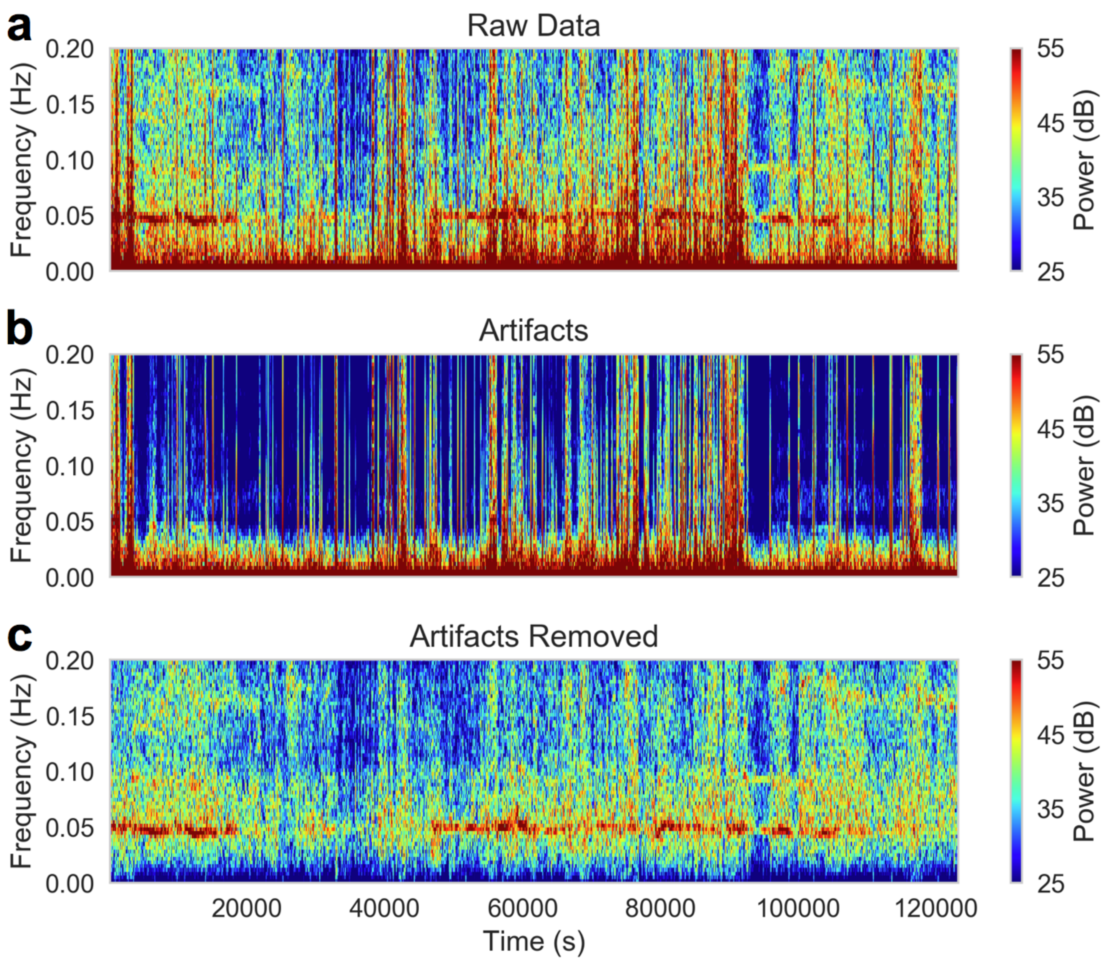

Supplementary Figure S1. (a) Spectrogram of the raw EGG signal for a representative ambulatory recording with motion artifacts visible as red vertical bands. (b) Spectrogram of the output of the LMMSE method, which captures motion artifacts and low frequency drift below 0.02 Hz while preserving the EGG signal near 0.05 Hz. (c) Spectrogram of the LMMSE output subtracted from the raw data, which reveals the artifact free EGG signal.
